# Dehydroepiandrosterone Stimulates Nerve Growth Factor and Brain Derived Neurotrophic Factor in Cortical Neurons

**DOI:** 10.1155/2013/506191

**Published:** 2013-12-04

**Authors:** Anahita Rahmani, Alireza Shoae-Hassani, Peyman Keyhanvar, Danial Kheradmand, Amir Darbandi-Azar

**Affiliations:** ^1^Stem Cell and Tissue Engineering Department, Research Center for Science and Technology in Medicine (RCSTiM), Tehran University of Medical Sciences, Tehran 19988-96953, Iran; ^2^Applied Cell Sciences Department, School of Advanced Technologies in Medicine, Tehran University of Medical Sciences, Tehran 19988-96953, Iran; ^3^Rajaie Cardiovascular, Medical and Research Centre, Iran University of Medical Sciences, Tehran 19969-14151, Iran; ^4^Faculty of Medicine, Islamic Azad University of Mashhad, Mashhad 91779-48564, Iran

## Abstract

Due to the increasing cases of neurodegenerative diseases in recent years, the eventual goal of nerve repair is very important. One approach for achieving a neuronal cell induction is by regenerative pharmacology. Nerve growth factor (NGF) and brain derived neurotrophic factor (BDNF) are neurotrophins that play roles in neuronal development, differentiation, and protection. On the other hand, dehydroepiandrosterone (DHEA) is a neurosteroid which has multiple actions in the nervous system. DHEA could be an important agent in regenerative pharmacology for neuronal differentiation during tissue regeneration. In this study, we investigated the possible role of DHEA to modulate NGF and BDNF production. The *in vivo* level of neurotrophins expression was demonstrated by ELISA in rat harvested brain cortex. Also neurotrophins expression after DHEA treatment was revealed by the increased neurite extension, immunostaining, and BrdU labeling in rats. Anti-NGF and anti-BDNF antibodies were used as suppressive agents on neurogenesis. The results showed that NGF and BDNF are overproduced after DHEA treatment but there is not any overexpression for NT-3 and NT-4. Also DHEA increased neurite extension and neural cell proliferation significantly. Overall, DHEA might induce NGF and BDNF neurotrophins overproduction in cortical neurons which promotes neural cell protection, survival, and proliferation.

## 1. Introduction

The central nervous system (CNS) is composed of an orchestrated control of cell proliferation, motility and maturation of neuronal and glial cells, axonal growth, neurite outgrowth, and the design of synapses. Neurotrophins are originally identified as important peptides involved in the development of nervous system and could determine neuronal differentiation phenotype. The neurotrophins that influence neural development include nerve growth factor (NGF), brain derived neurotrophic factor (BDNF), neurotrophin-3 (NT-3), NT-4/5, and neurotrophin-6 (NT-6) [1, 2]. Nerve growth factor (NGF) is the most important target-derived trophic factor for basal forebrain cholinergic neurons (BFCNs) [3]. These are small proteins, which share more than 50% sequence homology. These factors could enhance survival, proliferation, and differentiation of postmitotic neurons [4]. It is known that they could increase in neuronal numbers and neurite outgrowth [5]. So it is important to find molecules that promote overproduction of the neurotrophins. In this study, we focused to understand the induction of NGF and BDNF through dehydroepiandrosterone (DHEA) as a pharmacological agent.

DHEA is an adrenal, glial, and neuronal derived steroid. Although DHEA is produced by the human adrenal, it is not produced by the rodent adrenal. It has multiple actions in the nervous system but no specific receptor has been reported for this neurosteroid. DHEA could be an important agent in neuronal differentiation during development [6] or could provide a microenvironment for stem cells neurogenesis [7]. DHEA is present in very low concentrations in the blood of rats; however, the rodent brain may be able to make it from its precursor pregnenolone [6]. In adults, DHEA could act as anticorticosteroid molecule on *in vitro* cultures of neurons [8]. It protects hippocampal cells from oxidative stress [9] and antagonizes the neurotoxic effects of corticosterones in primary cultures of neurons [10].

The successful regeneration of the neurons is dependent on the cells survival and their progenitors proliferation [11]. From the point that DHEA (its sulfate form; DHEAS) is the most frequent neurosteroid in the human body, we hypothesized that DHEA may influence the NGF and BDNF production to induce neurogenesis and/or neuronal survival.

## 2. Materials and Methods

### 2.1. *In Vivo* Studies

#### 2.1.1. Animals Handling

This study was carried out in accordance with the Guide for the Care and Use of Laboratory Animals of the Tehran University of Medical Sciences. The protocol was approved by the Institutional Animal Care and Use Committee at the Research Center for Science and Technology in Medicine, Tehran University of Medical Sciences. Fifty-four male Wistar rats (aged between 15 and 45 days) were purchased from Pasteur institute, Tehran, Iran. The animals were housed in the polypropylene cages, three per cage, in a controlled temperature (22°C), under a 12 h light: dark cycle. Food and water were available *ad libitum*. We divided the animals into 6 treatment groups (three groups for *in vivo* studies and three groups just for extraction of cortical neurons); each contained 9 members. For *in vivo* studies, each group was subjected for DHEA treatment, BrdU labeling, DHEA measurement, and neurotrophins quantitation in triplicate.

#### 2.1.2. Drug Treatment

DHEA was used in the concentrations of 8 mg/kg daily subcutaneously under anesthesia for 2 weeks. The treatment dose was chosen according to several studies conducted on adult rats. This dose is in the middle of the range found effective in many studies and that might show oversensitivity to the higher doses [12–15].

#### 2.1.3. Bromodeoxyuridine Assay

Bromodeoxyuridine (BrdU) incorporation was assessed as described by Pechnick et al., in 2008 [16]. The rats were injected every 2 h with BrdU (Sigma-Aldridge, 100 mg/kg/i.p.) for a total of three injections and then sacrificed 24 h after the first BrdU injection. The entire cortex of the brain was cut into sections and processed using a BrdU Detection Kit (Roche Applied Biosystems, USA). Every third section (over 36 sections) was counted and the sum was multiplied by 3 to estimate the total number of BrdU-positive cells in the cortex region.

#### 2.1.4. DHEA Determination

DHEA basal level in brain was measured using Radioimmunoassay (RIA) kit (Diagnostic Systems Laboratories, USA). One mL of cortex region homogenates was extracted with 1 mL di-ethyl-ether, centrifuged at 300 g for 10 min, and kept for about 15 min at −70°C to freeze. The ether phase was decanted into a new glass tube, evaporated till dryness, and dissolved in 120 *μ*L of standard zero of the RIA kit. One hundred *μ*L of the solution was used for the determination of DHEA [17]. Cross-reactivity with other steroids is <0.2%. Results are presented in pmol/mg.

#### 2.1.5. Neurotrophic Factors Determination

The levels of NGF, BDNF, NT-3, and NT-4 were determined using Emax ImmunoAssay System (Promega, USA) as previously described [18]. Briefly the brain cortex from Wistar rats were collected in homogenization buffer (1 : 10 diluted, 0.4 M NaCl, 5 mM EDTA, 0.5% bovine serum albumin (BSA), 1 mM phenylmethylsulfonyl fluoride (PMSF), and 0.1% Triton X-100) and sonicated for 15 s in an ice bath. After the supernatant was collected in a new tube, the pellet was carefully washed twice with phosphate buffered saline (PBS) and resuspended in cold homogenization buffer. Each fraction was assayed in parallel ELISA Emax ImmunoAssay System (Promega, USA).

### 2.2. *In Vitro* Studies

#### 2.2.1. Isolation and Culture of Cortical Neurons

Cortical neurons were isolated from cerebral cortex of rats. Isolated cerebral cortex was suspended in neurobasal medium (GIBCO, USA) containing B-27 supplement (Invitrogen, USA). The suspension was placed on poly-D-lysine coated plates. The cultures were kept in the 90% humidity and 10% CO_2_ atmosphere at 37°C for 14 days and media were half-replaced with fresh media every 3 days.

#### 2.2.2. DHEA Treatment

DHEA was used as a series of concentration (10^−4^–10^−8^ mol/mL) in neurobasal medium. Cortical neurons were isolated from cerebral cortex of rats suspended in this treated medium. The cultures were kept in the 90% humidity and 10% CO_2_ atmosphere at 37°C for 2 weeks as described above. Anti-NGF and anti-BDNF antibodies (each 2 *μ*g/mL, Santa Cruz, CA, USA) were used to block NGF and BDNF action from DHEA treated neurons. A group from each treatment was kept for one month (30 days) to survey the cell survival after treatments. MTT assay was used to determine the survival rate of cells before and after treatment.

#### 2.2.3. ELISA

The amount of released neurotrophins was quantified from medium of treated neuron (Promega kit, USA). ELISA was performed according to the manufacturer's manual. Briefly, the wells of plates were coated with anti-NGF/anti-BDNF mAb diluted with carbonate coating buffer at 4°C for 24 h. After 1 h blockade with blocking buffer, the plates were incubated with standard and culture medium sample for 2 h followed by incubation with anti-rat BDNF/anti-rat NGF polyclonal Ab. After 1 h incubation of Anti-Ig HRP conjugate, the reaction was developed with tetra-methyl-benzidine and the absorbance was read at 450 nm with a plate reader after stopping the reaction with 1 N HCl.

#### 2.2.4. Immunocytochemistry Assay

Isolated cortical neurons from treated rats in all groups were plated on 24-well plates (Greiner, Germany). These cells were fixed in 4% paraformaldehyde (PFA in PBS) and washed in PBS. The cells were then incubated in 2 M HCl for 30 min at 37°C. Then they was neutralized with 0.1 M NaOH and washed several times in PBS. Fixed cells were blocked in 5% bovine serum albumin (BSA) with 0.3% Triton X-100 and incubated with anti-MAP-2 (microtubule associated protein 2) antibody (monoclonal 1 : 1000, Chemicon). After incubation with the primary antibodies, FITC conjugated secondary antibody (1 : 500, AbCam, UK) was used to visualize the signal with Ceti immunofluorescence microscopy [19].

#### 2.2.5. Statistical Analysis

Data are presented as means ± SD. DHEA and BDNF basal levels were analyzed by independent *t*-tests. Significance of differences was tested by ANOVA. Differences were considered significant at values of *P* ≤ 0.05.

## 3. Results

### 3.1. DHEA Stimulated NGF and BDNF Expression and Release

**Figure 1:**
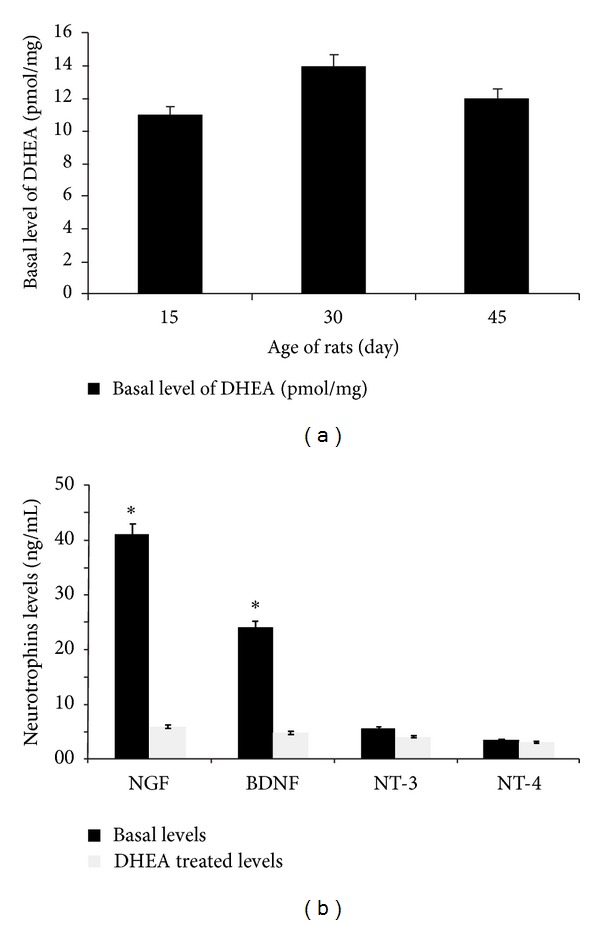
Mean basal levels of DHEA of 15–30–45-day old Wistar rats (a). Mean basal levels of NGF, BDNF, NT-3, and NT-4 neurotrophins in the brain cortex of 30-day old Wistar rats and Mean levels of neurotrophins after DHEA treatment in the brain cortex of Wistar rats. A comparison between basal levels of NGF, BDNF, NT-3, and NT-4 neurotrophines before and after DHEA treatment in the brain cortex of Wistar rats. The levels of NGF, BDNF, NT-3, and NT-4 neurotrophines were measured using ELISA (b). In each part, the data are obtained from nine animals.

The basal levels of DHEA (Figure 1(a)), NGF, and BDNF in the brain cortex are presented in Figure 1(b). ELISA results showed that treatment of DHEA increased NGF and BDNF levels, 7- and 5-fold (Figure 1(b)) compared with control, respectively (Figure 1(b)). The levels of NT-3 and NT-4 did not influence after DHEA treatment (Figure 1(b)).

### 3.2. DHEA Stimulated Cortical Neurons Proliferation

**Figure 2:**
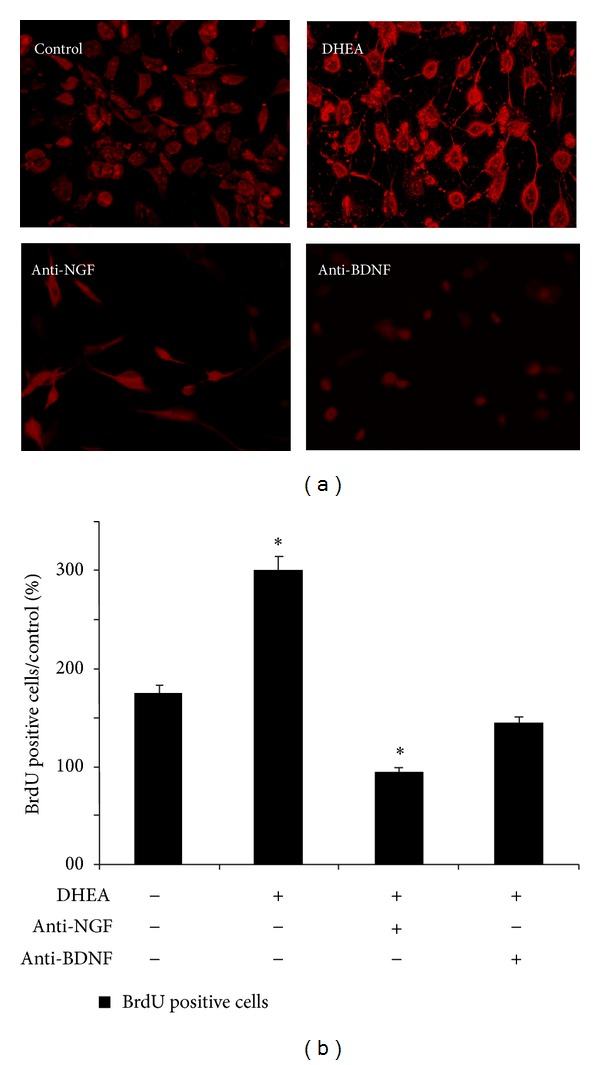
Number of BrdU+ cells. Representative image showing BrdU+ cells in the brain cortex under DHEA treatment and using antineurotrophins after DHEA treatment (a). The majority of BrdU+ cells which survive in brain cortex of Wistar rats (b). In each experiment, the data were obtained from nine animals.

Rats treated with DHEA daily showed increase in neural cells proliferation. BrdU-labeled neurons were found in all treatment groups (Figure 2(a)). Analysis by one-way ANOVA also showed a significant effect of treatment on neurogenesis. DHEA increased the number of BrdU-labeled cells (Figure 2(b)). Antineurotrophin antibodies were decreased in the BrdU-labeled cells. To test the involvement of NGF and BDNF in the proliferation effect of DHEA, we used their antibodies to inactivate these neurotrophins. Anti-NGF and anti-BDNF decreased the neurogenesis even more than DHEA nontreated groups. Among these 2 neurotrophic factors the role of NGF was more important than BDNF (Figure 2(b)). The number of newly formed cells was higher in the rats just received DHEA. Also there were more neuronal cells in the group received anti-BDNF in comparison with those received anti-NGF. However there must be a relationship between these neurotrophins amount and function.

### 3.3. DHEA Induces Neurite Outgrowth in Cultured Rat Neurons

**Figure 3:**
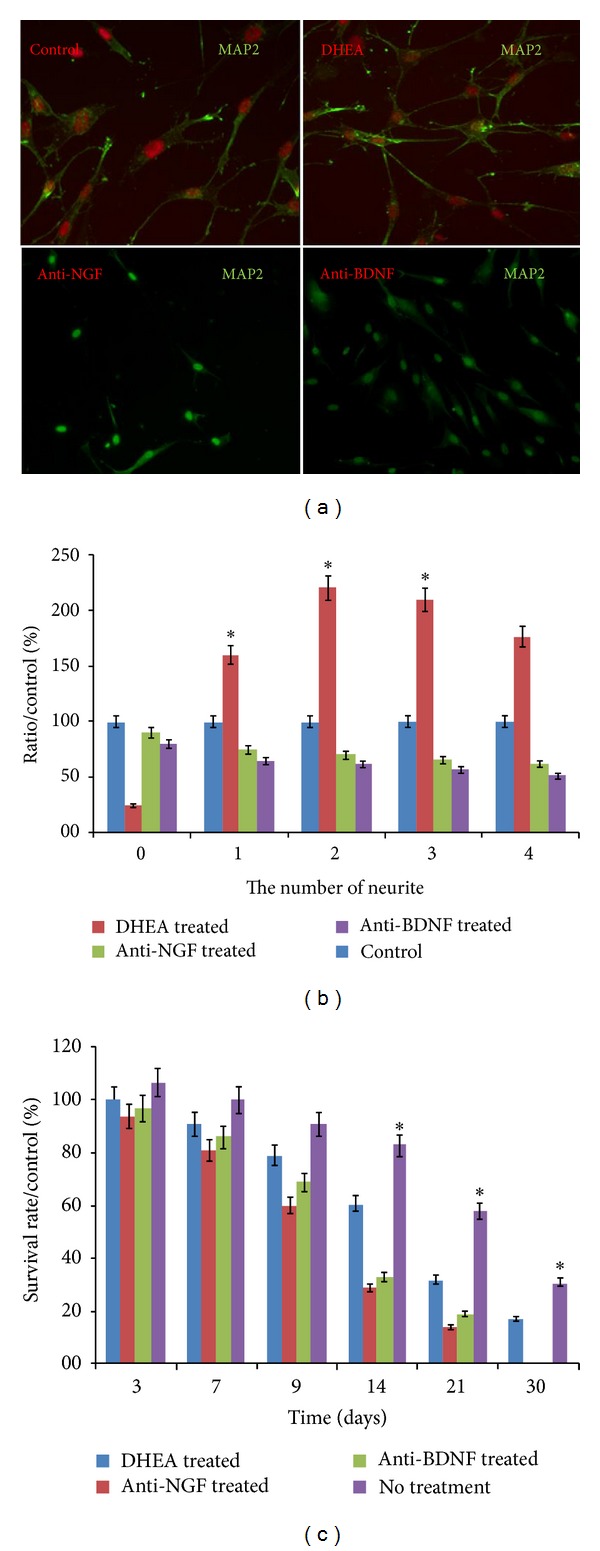
DHEA induced upregulation of NGF and BDNF facilitated the differentiation of rat cortical neurons. Neurons from the DHEA treated rats were immunostained against neuronal marker MAP-2 (a). The number of neurite extending from each neuron is demonstrated. Anti-NGF and anti-BDNF antibodies (1 *μ*g/mL) were used to block NGF and BDNF action from DHEA treated groups (b). Effects of chronic DHEA treatment on cell proliferation and survival via MTT assay (c). Data represent the mean ± S.D. In each experiment, the data were obtained from nine animals.

Considering the roles of NGF and BDNF in neurons, we investigated the role of DHEA on the neurite outgrowth in rat cortical neurons. The cultured cortical neurons were treated with DHEA and visualized by a neural cell marker, MAP-2 (Figure 3(a)). DHEA increased the average number of neurite branches extending from a single neuron as well as the length of individual neurite (Figure 3(b)). Treatment with anti-NGF inhibited neurite outgrowth significantly. Anti-BDNF Ab inhibited neurite outgrowth too, but this effect was lower than that of anti-NGF Ab (Figure 3(b)). Overall, the results suggest that DHEA enhances neurite outgrowth by regulating NGF and BDNF expression and release.

### 3.4. DHEA Induces Longer-Term Survival in Cultured Neurons

The rat cortical neuron cultures treated with DHEA maintained for one month in incubator refreshing the media every 3 days. Extending DHEA treatment throughout the 30 d survival period increased the numbers of viable cells (Figure 3(c)). One-way ANOVA followed by pairwise comparisons showed that DHEA by itself increased the number of neuronal cells (presumed neurons) compared to controls (no DHEA) (Figure 3(c)).

## 4. Discussion

In this study, we provided evidences that DHEA increased NGF and BDNF production, neuronal cell proliferation, neuronal cell survival, and neurite outgrowth. The microenvironment of the CNS plays a major role in controlling neurogenesis. Levels of neurosteroids in the blood and/or CNS are a significant determinant in the formation of new neuronal cells. Our results add a new point to this map: the neurosteroid DHEA promotes overexpression of NGF and BDNF.

DHEA is an important regulator for the proliferation of neural stem cells [20–22]. First, we have determined the basal levels of DHEA, NGF, BDNF, NT-3, and NT-4 molecules in the brain cortex segments of Wistar rats (Figures 1(a) and 1(b)). DHEA (10^−6 ^mol/liter) administered to animals for 2 weeks increased the number of newly formed cells in the brain cortex in samples that harvested 24 h (Figure 2(b)) after the last injection of BrdU. In this time, we have the elevation rate of NGF and BDNF production significantly observed (Figure 1(b)) but there were no significant increase in NT-3 and NT-4 levels (Figure 1(b)). The numbers of labeled cells receiving DHEA and anti-NGF or anti-BDNF even was smaller than that of controls (Figures 2(a) and 2(b)). The cytotoxic effect of DHEA alone or with BDNF was not significant in concentrations 10^−6^ or lower (data not presented here).

We showed that DHEA in the absence of NGF and BDNF did have a little effect on neurite outgrowth, but activation of the cells by NGF and BDNF is necessary to the increase of neuronal specific biomarkers (Figure 3(a)) and neurite outgrowth and expression (Figure 3(b)).

Despite the Ziegler results that showed that DHEA decreases NGF-induced cell survival and shifts the cells toward a neuroendocrine phenotype [23], our results showed the increasing survival rate of DHEA treated cells in the concentration that has no cytotoxic effect on brain cortex cells. On the other hand the cell source in our experiment was different from that of them.

Activation of cells by NGF and/or BDNF is necessary for DHEA to mediate the neurite outgrowth (Figures 3(a) and 3(b)). In a study by Compagnone and Mellon, they have demonstrated that DHEA in low concentrations of 10^−9 ^mol/liter caused neurite outgrowth in primary cultures of mouse embryonic neurons [6]. In this case, our results confirm their experiment; even we have different culture conditions and different cell type from that used by Compagnone and Mellon.

The steroids amount in the blood or brain is sensitive to a variety of external events or intrinsic processes. So, the steroid dependent control is a significant determinant of variations in the differentiation or the formation of new cells. In 2000, Åberg et al. showed that DHEA may activate the expression of growth factors like systemic IGF that are contributing in neurogenesis [23]. Also Morales et al. demonstrated that DHEA increases the plasma concentration of these factors [24] and that decrease in DHEA plasma levels is associated with neural cells degeneration [25]. Our data showed the dose dependent activity of DHEA (data not shown). The higher concentration (10^−4 ^mol or higher) was cytotoxic for our cells. On the contrary, other groups showed that DHEA could protect neurons from amino acid induced cytotoxicity [8]. So the effect of DHEA varies between the different cells. Also our experiments showed increase in MAP-2 expression after the treatment with DHEA (Figure 3(a)). Administration of anti-NGF and anti-BDNF antibodies shows that DHEA induces MAP-2 in cells via the formation of NGF and BDNF. If the newly formed cells under DHEA treatment resembled those formed neurons under basal conditions, then our work suggests that regenerative pharmacology could provide that they will become part of the functional neural network.

The neurotrophins exert both long-term cell survival effects and short-term effects on neuronal cells. It is confirmed that BDNF promotes long-term effects on neurons [26]. Our results demonstrated the significant increase in survival time of DHEA treated neurons especially after 2 weeks (Figure 3(c)). It appears that NT-3 is regulated independent of neuronal activity, but its secretion is dependent on hormonal levels and on BDNF levels [27]. Our results showed insignificant changes in NT-3 and NT-4 secretion. According to Lindholm et al., NT-3 indirectly can also be affected by the BDNF levels in the brain. So the DHEA has no direct effect on these neurotrophins (NT-3 and NT-4).

In a study by Gubba et al., they showed that DHEA (100 nM) upregulated NGF after 3 h, but not at other time points (12, 24, or 48 h). Also they showed that DHEA had no effect on the other neurotrophins in mixed primary cultures [28]. Of course they used different concentrations of DHEA and astrocytes in their study.

Another hypothesis that has an important part in our conclusion is that DHEA could bind to the NGF and BDNF receptors and in this way exert these effects. Lazaridis et al. have shown that DHEA binds to the NGF receptor [29]. So it is possible that BDNF could provide the receptors for DHEA too. The decreased levels of NGF and BDNF are found to decrease trkA receptor and a loss of cholinergic neurons [30]. The pharmacologically induced strengthening of the cholinergic system may play a role in alleviating age-associated dysfunctions.

## 5. Conclusion

DHEA stimulates NGF and BDNF neurotrophins overexpression and release. It enhances neuronal cell survival, neuronal cell proliferation, and neurite outgrowth via these neurotrophins. If our results could be generalized for human, so formation of new cells in the brain via regenerative pharmacology could be important in treating neurodegenerative diseases such as Alzheimer and Parkinson.

## References

[1] Berkemeier LR, Winslow JW, Kaplan DR, Nikolics K, Goeddel DV, Rosenthal A (1991). Neurotrophin-5: a novel neurotrophic factor that activates trk and trkB. *Neuron*.

[2] Noureddini M, Verdi J, Mortazavi Tabatabaei SA, Sharif S, Shoae-Hassani A (2012). Human endometrial stem cell neurogenesis in response to NGF and bFGF. *Cell Biology International*.

[3] Levi-Montalcini R (1987). The nerve growth factor: thirty-five years later. *The EMBO Journal*.

[4] Davies AM (1994). The role of neurotrophins in the developing nervous system. *Journal of Neurobiology*.

[5] Ahmed S, Reynolds BA, Weiss S (1995). BDNF enhances the differentiation but not the survival of CNS stem cell-derived neuronal precursors. *The Journal of Neuroscience*.

[6] Compagnone NA, Mellon SH (1998). Dehydroepiandrosterone: a potential signalling molecule for neocortical organization during development. *Proceedings of the National Academy of Sciences of the United States of America*.

[7] Shoae-Hassani A, Mortazavi-Tabatabaei SA, Sharif S, Rezaei-Khaligh H, Verdi J (2011). DHEA provides a microenvironment for endometrial stem cells neurogenesis. *Medical Hypotheses*.

[8] Kimonides VG, Khatibi NH, Svendsen CN, Sofroniew MV, Herbert J (1998). Dehydroepiandrosterone (DHEA) and DHEA-sulfate (DHEAS) protect hippocampal neurons against excitatory amino acid-induced neurotoxicity. *Proceedings of the National Academy of Sciences of the United States of America*.

[9] Bastianetto S, Ramassamy C, Poirier J, Quirion R (1999). Dehydroepiandrosterone (DHEA) protects hippocampal cells from oxidative stress-induced damage. *Molecular Brain Research*.

[10] Kimonides VG, Spillantini MG, Sofroniew MV, Fawcett JW, Herbert J (1999). Dehydroepiandrosterone antagonizes the neurotoxic effects of corticosterone and translocation of stress-activated protein kinase 3 in hippocampal primary cultures. *Neuroscience*.

[11] Chopp M, Li Y (2002). Treatment of neural injury with marrow stromal cells. *The Lancet Neurology*.

[12] Durand M, Aguerre S, Fernandez F (2000). Strain-dependent neurochemical and neuroendocrine effects of desipramine, but not fluoxetine or imipramine, in Spontaneously Hypertensive and Wistar-Kyoto rats. *Neuropharmacology*.

[13] Tejani-Butt S, Kluczynski J, Paré WP (2003). Strain-dependent modification of behavior following antidepressant treatment. *Progress in Neuro-Psychopharmacology and Biological Psychiatry*.

[14] Will CC, Aird F, Redei EE (2003). Selectively bred Wistar-Kyoto rats: an animal model of depression and hyper-responsiveness to antidepressants. *Molecular Psychiatry*.

[15] Malkesman O, Asaf T, Shbiro L (2009). Monoamines, BDNF, dehydroepiandrosterone, DHEA-Sulfate, and childhood depression: an animal model study. *Advances in Pharmacological Sciences*.

[16] Pechnick RN, Zonis S, Wawrowsky K, Pourmorady J, Chesnokova V (2008). p21Cip1 restricts neuronal proliferation in the subgranular zone of the dentate gyrus of the hippocampus. *Proceedings of the National Academy of Sciences of the United States of America*.

[17] Maayan R, Morad O, Dorfman P, Overstreet DH, Weizman A, Yadid G (2005). The involvement of dehydroepiandrosterone (DHEA) and its sulfate ester (DHEAS) in blocking the therapeutic effect of electroconvulsive shocks in an animal model of depression. *European Neuropsychopharmacology*.

[18] Barde YA, Edgar D, Thoenen H (1982). Purification of a new neurotrophic factor from mammalian brain. *The EMBO Journal*.

[19] Rahmani A, Kheradmand D, Keyhanvar P, Shoae-Hassani A, Darbandi-Azar A (2013). Neurogenesis and increase in differentiated neural cell survival via phosphorylation of Akt1 after fluoxetine treatment of stem cells. *BioMed Research International*.

[20] Karishma KK, Herbert J (2002). Dehydroepiandrosterone (DHEA) stimulates neurogenesis in the hippocampus of the rat, promotes survival of newly formed neurons and prevents corticosterone-induced suppression. *European Journal of Neuroscience*.

[21] Suzuki M, Wright LS, Marwah P, Lardy HA, Svendsen CN (2004). Mitotic neurogenic effects of dehydroepiandrosterone (DHEA) on human neural stem cell cultures derived the fetal cortex. *Proceedings of the National Academy of Sciences of the United States of America*.

[22] Shoae-Hassani A, Sharif S, Verdi J (2011). The neurosteroid dehydroepiandrosterone could improve somatic cell reprogramming. *Cell Biology International*.

[23] Åberg MAI, Åberg ND, Hedbäcker H, Oscarsson J, Eriksson PS (2000). Peripheral infusion of IGF-I selectively induces neurogenesis in the adult rat hippocampus. *The Journal of Neuroscience*.

[24] Morales AJ, Nolan JJ, Nelson JC, Yen SSC (1994). Effects of replacement dose of dehydroepiandrosterone in men and women of advancing age. *Journal of Clinical Endocrinology and Metabolism*.

[25] Charalampopoulos I, Alexaki V, Tsatsanis C (2006). Neurosteroids as endogenous inhibitors of neuronal cell apoptosis in aging. *Annals of the New York Academy of Sciences*.

[26] Korte M, Kang H, Bonhoeffer T, Schuman E (1998). A role for BDNF in the late-phase of hippocampal long-term potentiation. *Neuropharmacology*.

[27] Lindholm D, Castren E, Berzaghi M, Blochl A, Thoenen H (1994). Activity-dependent and hormonal regulation of neurotrophin mRNA levels in the brain: implications for neuronal plasticity. *Journal of Neurobiology*.

[28] Gubba EM, Fawcett JW, Herbert J (2004). The effects of corticosterone and dehydroepiandrosterone on neurotrophic factor mRNA expression in primary hippocampal and astrocyte cultures. *Molecular Brain Research*.

[29] Lazaridis I, Charalampopoulos I, Alexaki V (2011). Neurosteroid dehydroepiandrosterone interacts with nerve growth factor (NGF) receptors, preventing neuronal apoptosis. *PLoS Biology*.

[30] Pham TM, Ickes B, Albeck D, Söderström S, Granholm A-C, Mohammed AH (1999). Changes in brain nerve growth factor levels and nerve growth factor receptors in rats exposed to environmental enrichment for one year. *Neuroscience*.

